# Pre- and Postoperative Circulating IGF-I, IGFBP-3, and IGFBP-7 Levels in Relation to Endocrine Treatment and Breast Cancer Recurrence: A Nested Case-Control Study

**DOI:** 10.3389/fonc.2021.626058

**Published:** 2021-03-09

**Authors:** Ann H. Rosendahl, Sofie Björner, Maria Ygland Rödström, Karin Jirström, Signe Borgquist, Christian Ingvar, Michael N. Pollak, Helena Jernström

**Affiliations:** ^1^ Department of Clinical Sciences Lund, Oncology, Lund University and Skåne University Hospital, Lund, Sweden; ^2^ Department of Clinical Sciences Lund, Oncology and Therapeutic Pathology, Lund University and Skåne University Hospital, Lund, Sweden; ^3^ Department of Oncology, Aarhus University and Aarhus University Hospital, Aarhus, Denmark; ^4^ Department of Clinical Sciences Lund, Surgery, Lund University and Skåne University Hospital, Lund, Sweden; ^5^ Lady Davis Institute for Medical Research, Jewish General Hospital and Department of Oncology McGill University, Montreal, QC, Canada

**Keywords:** IGF-I, IGFBP-3, IGFBP-7, IGF-IR, breast cancer, recurrence

## Abstract

Insulin-like growth factor-I (IGF-I) and its binding proteins (BPs) have been associated with breast cancer risk, especially high IGF-I concentrations and the biologically active fraction estimated as the IGF-I/IGFBP-3 molar ratio. The relation of circulating IGF-I and IGFBP-3 concentrations with risk of breast cancer recurrence has been less documented. In addition a new member to a sub-group of the IGFBP-superfamily was recently identified, the low affinity IGFBP-7. To date, the role of systemic IGFBP-7 in breast cancer progression has not been investigated. Our purpose was to establish whether circulating IGF-I, IGFBP-3, and IGFBP-7 levels are related to recurrence-risk in breast cancer. A case-control study was nested within the population-based BCBlood cohort of 853 breast cancer patients diagnosed 2002–2010 in Sweden and followed through 2012. In total, 95 patients with recurrence and 170 controls were matched on age and tumor characteristics. Plasma IGF analytes and tumor membrane IGF-I receptor (IGF-IR^m^) positivity were analyzed and recurrence-risk was evaluated with conditional logistic regression. Preoperative tertiles of IGF-I and IGFBP-3 were both positively associated with recurrence-risk, but not IGFBP-7. The trend was of borderline significance for IGF-I, T1:REF, T2 OR:1.6, T3 OR: 2.2 adjusted *P*_trend_=0.057 and significant for IGFBP-3 T1:REF, T2 OR:1.2, T3 OR: 2.1 adjusted *P*_trend_=0.042. The models were adjusted for age, anthropometric factors, smoking, and treatments. There was a significant interaction between IGFBP-7 and IGF-IR^m^ positivity on recurrence, where the highest IGFBP-7 highest IGFBP-7 tertile conferred increased recurrence-risk in patients with IGF-IR^m^ positive tumors but not in those with IGF-IR^m^ negative tumors (*P*_interaction_=0.024). By the 1-year visit, age-adjusted IGF-I levels were reduced by 17% while IGFBP-3 and IGFBP-7 were stable. IGF-I levels were significantly reduced by radiotherapy in all patients and by tamoxifen in patients with ER^+^ tumors. Postoperative changes >10% (n=208) in IGF-I, IGFBP-3, IGFBP-7, or the IGF-I/IGFBP-3 ratio did not predict recurrence after adjustment for preoperative levels, age, anthropometric factors, smoking, and treatments. In conclusion, this study suggests that preoperative IGF-I and IGFBP-3 levels, but not postoperative changes, might provide independent prognostic information and influence breast cancer recurrence. The role of IGFBP-7 in breast cancer merits further study.

## Introduction

Breast cancer is the most common type of cancer among women and with novel diagnostic and therapeutic modalities is often a treatable disease. Still, for women diagnosed with metastatic or recurrent breast cancer, the prognosis is poor. Means to better predict clinical outcome and to identify women at risk of breast cancer recurrence may be helpful to optimize individual treatment decisions and improve prognosis.

Insulin-like growth factor I (IGF-I) has a well-established role as a mitogenic peptide growth factor and has been suggested in multiple studies to be associated with predisposition of several types of cancer (1, 2). IGF-I can affect epithelial cell proliferation and reduce apoptosis *via* activation of the type I IGF receptor (IGF-IR). An early groundbreaking clinical report demonstrated that pre-menopausal women with higher circulating IGF-I levels (top tertile) had an increased risk of breast cancer, compared with patients with lower levels (bottom tertile) (3). Subsequent reports have supported a positive association between systemic IGF-I levels and breast cancer risk among pre-menopausal, but also among post-menopausal women (4–6). The compelling evidence from pre-clinical reports and epidemiology studies of a tumor promoting role by the IGF-I/IGF-IR axis translated into several clinical development programs targeting the IGF-IR. Although some early promising reports, large phase III clinical trials have not shown clear clinical benefit, which may relate to the complexity of the IGF system, suboptimum dosing of drug candidates, as well as inadequate patient selection of probable responders, as reviewed in (7).

The majority of systemic IGF-I (>90%) circulates in complex with IGFBP-3, one of six classic high affinity IGF binding proteins (IGFBPs) (8). These complexes increase the half-life of IGF-I while reducing its bioavailability for activating the IGF-IR. Besides its IGF binding properties, IGFBP-3 also has IGF-independent actions both through cell surface interactions and nuclear translocation (9). Early studies indicated predominantly growth inhibitory actions by IGFBP-3, where it e.g., can induce apoptosis *via* interaction with the nuclear retinoid X receptor α (RXR-α) (10). In addition, both pre-clinical and clinical reports demonstrate tumor-promoting effects by IGFBP-3 (11, 12). High expression of IGFBP-3 is found in aggressive breast cancer, and IGFBP-3 has been shown to potentiate epidermal growth factor receptor signaling in triple negative breast cancer (13–15). IGFBPs are also known to exert both IGF-dependent and independent actions (9, 16, 17), with distinct functions in the mammary gland (18). The relation of circulating IGF-I and IGFBP-3 concentrations with risk of breast cancer recurrence has been less documented.

More recently, a new member to a sub-group of the IGFBP-superfamily was identified, the low affinity IGFBP-7 [also known as IGFBPrp1/mac25/prostacyclin-stimulating factor (PSF), tumor adhesion factor (TAF), and angiomodulin (AGM)] (8, 19). IGFBP-7 appears to be a pleiotropic protein displaying contrasting roles dependent on cell phenotype or context. It also stimulates prostacyclin production and cell adhesion. Previous studies have demonstrated that IGFBP-7 interacts with unbound IGF-IR at the cell surface and prevents or inhibit downstream signaling (20). IGFBP-7 has also been shown to bind insulin with high avidity (21). When IGFBP-7 is bound to the ligands for insulin and IGF-I receptors in breast cancer cells, IGFBP-7 neutralizes mitogenic signaling and induces senescence (8). However, while a tumor suppressive role of IGFBP-7 has been shown for epithelial-like tumor cells, others have shown tumor promoting properties and stimulation of anchorage-independent growth in epithelial cells with an epithelial-to-mesenchymal (EMT) phenotype and in malignant mesenchymal cells (22). In human breast cancer tissue, down-regulation of IGFBP-7 was associated with down-regulation of retinoblastoma protein, cyclin E overexpression, and impaired prognosis (23). To date, the role of systemic IGFBP-7 in breast cancer progression has not been investigated.

A more complete understanding of IGF-I and IGFBP system is necessary to determine their prognostic and treatment predictive value, especially since the impact of changes in analyte levels post-surgery and during treatment remains unclear. We hypothesized that we would observe an increased risk of breast cancer recurrence with elevated IGF-I and IGFBP-3 levels while elevated IGFBP-7 may confer better prognosis. The aim of the present nested case-control study was to examine associations between pre- and postoperative circulating IGF-I, IGFBP-3 and IGFBP-7 levels, and risk of breast cancer recurrence.

## Materials and Methods

### Study Population

**Figure 1:**
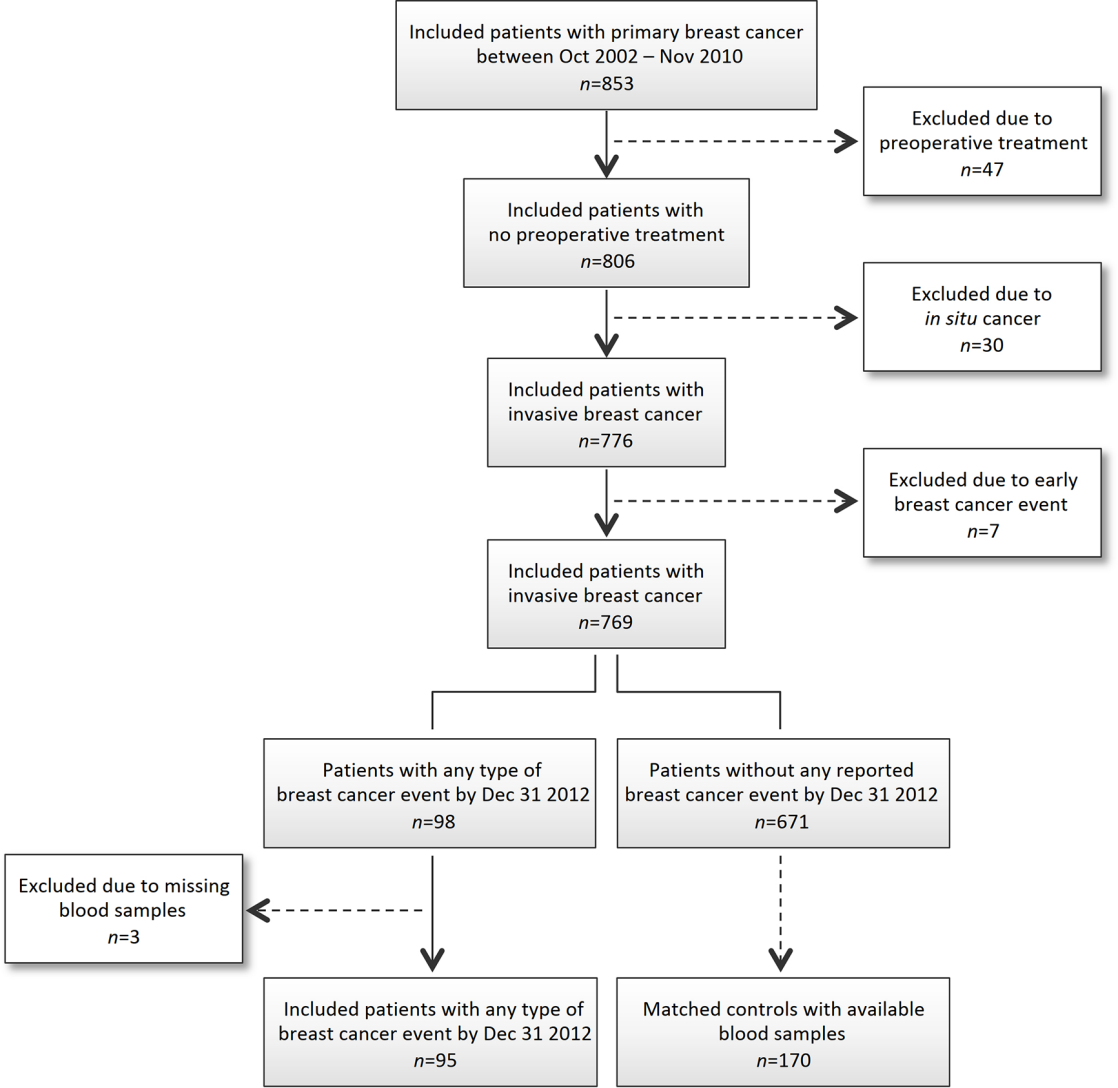
Flowchart of included and excluded patients.

This nested case-control study was based on women with breast cancer included in the ongoing population-based Lund BC Blood study. The present study population was identified among 853 patients in the BC blood cohort, aged 24 to 99 years, diagnosed with invasive primary breast cancer between October 2002 and November 2010 at the Skåne University Hospital (Lund, Sweden), without preoperative treatment and with no other cancer history within the past 10 years. A flow-chart of the selection process of patients are shown in **Figure 1**. Details of the BC Blood cohort have previously been outlined (24–26). At the preoperative visit patients filled out a 3-page questionnaire on lifestyle and a research nurse obtained their body measurements. All participants signed a written informed consent. The study was approved by the Lund Ethics committee (LU 75-02, 37-08, 658-09, 58-12, 379-12, 227-13, 277-15, 458-15).

### Nested Case-Control Selection

Cases, defined as patients with breast cancer recurrence during follow-up, and later than 3 months after their primary breast cancer diagnosis, were matched to recurrence-free controls by age at study inclusion (± 5 years), tumor ER status, lymph node involvement, and invasive tumor size. One case (aged 25 years) was matched to a 6-year older control as no control within the ±5 year time span was available. Breast cancer recurrence included local-, regional- or distant event, or contralateral/second primary breast cancer. Ninety-eight women within the BC blood cohort were identified with recurrence before January 1, 2013, of which three women were not included in the present study due to missing blood samples. For each case-subject, up to two controls were selected. The follow-up time for each control was longer than the time to recurrence for the matching case, and at least five years from diagnosis. Four patients with breast cancer event were matched to five controls with shorter follow-up than five years, but longer than their matched cases. These controls were recurrence-free and free of new breast cancer on February 28, 2014. Overall, 95 patients with breast cancer recurrence were matched to 170 controls (in total 265 women). Seventy-five patients were each matched to two controls, while for 20 patients only one control was available for matching.

### Measurements of IGF-I, IGFBP-3, and IGFBP-7 Plasma Levels

Blood samples were collected at the preoperative visit and at the 1-year postoperative follow-up visit, routinely processed and plasma aliquots frozen at −80°C within 2 h of sampling. All samples were coded and blinded to case or control status. Total IGF-I and IGFBP-3 plasma levels were measured using a magnetic microbead chemiluminescence technology (Immunodiagnostic Systems Inc., Boldon, UK). Total IGFBP-7 plasma levels were measured using ELISA (Antigenix America Inc., Huntington Station, NY). Case-control sets, ordered randomly, were analyzed together in the same assay batch, except for two controls that were analyzed in a batch different from their matched case. The IGF-I/IGFBP-3 molar ratio was computed based on the following equivalents for conversion: 1 ng/ml = 0.130 nmol/L for IGF-I and 0.036 nmol/L for IGFBP-3. The intra-assay and inter-assay coefficients of variation from replicate quality control samples were 3.15 and 4.15%, respectively, for IGF-I; 1.94 and 4.08% for IGFBP-3 and 6.52 and 10.44% for IGFBP-7.

### Immunohistochemical Evaluation of IGF-IR Membrane Expression in Primary Tumors

Tissue microarrays (TMAs) with duplicate 1 mm cores were constructed from representative tumor regions of formalin-fixed paraffin-embedded tissue blocks, as previously described (27). Immunohistochemical staining was performed on 4 µm deparaffinized and pre-treated TMA sections using an automatic immunostainer (TechMate™500 Plus, DAKO, Glostrup, Denmark), DAKO Envision, and anti-IGF-IRβ antibody (1:150, Santa Cruz Biotechnology, Cat. No.: sc-713). The IGF-IR membrane (IGF-IR^m^) expression was evaluated by two independent observers (AR, SBj) and scored as either negative or any positive membrane staining.

### Statistical Analyses

IGF-I, IGFBP-3, IGFBP-7 levels, and IGF-I/IGFBP-3 molar ratios were categorized into tertiles based on the distribution levels among control women. IGF-I levels ranged from 54 to 304 ng/ml and the cut-off values between tertiles were 110.1 and 149.6 ng/ml (T1 = 54–110 ng/ml, T2 = 111–149 ng/ml, T3 = 150–304 ng/ml). IGFBP-3 levels ranged from 2,006 to 6,832 ng/ml and the cut-off values between tertiles were 3,812 and 4,493 ng/ml (T1 = 2,006–3,811 ng/ml, T2 = 3,812–4,488 ng/ml T3 = 4,493–6,832 ng/ml). IGFBP-7 levels ranged from 18.6 to 184.8 ng/ml and the cut-off values between tertiles were 31.0 and 38.3 ng/ml (T1 = 18.6–30.9 ng/ml T2 = 31.0–38.1 ng/ml, T3 = 38.3–184.8 ng/ml). IGF-I/IGFBP-3 molar ratios ranged from 0.051 to 0.299 and the cut-off values between tertiles were 0.099 and 0.128 (T1 = 0.051–0.099 ng/ml, T2 = 0.100–0.128 ng/ml, T3 0.129–0.299 ng/ml).

Spearman rank correlation (R_s_) was used to assess the correlation between age and pre-operative analyte levels. Age-adjusted conditional logistic regression models were used to compare clinicopathological characteristics between cases and controls. Associations between clinicopathological characteristics and age adjusted circulating baseline hormone levels were determined using generalized linear means and presented as geometric means with 95% Wald confidence intervals (CI) for IGF-I, IGFBP-7 IGFBP-3, and IGF-I/IGFBP-3 molar ratio. To determine risk of breast cancer recurrence among the case-control sets according to tertile values of IGF-I, IGFBP-3, IGFBP-7 levels, or IGF-I/IGFBP-3 molar ratios, conditional logistic regression models adjusted for age were used to provide conditional odds ratios (OR) with 95% CI. The influence of IGFBP-7 levels in relation to IGF-IR membrane positive tumors on risk of breast cancer recurrence was analyzed using multivariable Cox regression to calculate OR with 95% CI, adjusted for age. Multivariable models were further adjusted for BMI ≥ 25 kg/m^2^, waist circumference ≥88 cm, preoperative smoking, and treatments prior to recurrence in the case and by the corresponding follow-up time in the matched control. Two interaction variables were calculated between IGFBP-7 T2 and T3 and IGF-IR^m^ positive tumor to assess potential effect modifications. Generalized linear mean with 95% Wald CI adjusted for age were used to assess percentage change from the pre- to postoperative values in relation to clinicopathological characteristics and treatments. Changes in analyte levels >10% from preoperative values (reduction >10%, stable max ±10%, or increase >10%) were assessed in relation to recurrence in conditional logistic regression models. All statistical analyses were performed using SPSS Statistics 24.0 software (IBM, Chicago, IL, USA). A *P*-value of <0.05 was considered statistically significant. Since this was an exploratory study, nominal *P*-values are presented without adjustment for multiple testing.

## Results

### Patient Characteristics

The median age difference between patients with breast cancer recurrence (“cases”) and matched recurrence-free controls within each stratum was 0.08 years (IQR −0.71–1.08). IGF-I (R_s_=−0.51), IGFBP-3 (R_s_= −0.31), and IGF-I/IGFBP-3 molar ratio (R_s_=−0.43) were strongly negatively correlated with age (all *P*_s_<0.0001). In contrast, IGFBP-7 was positively correlated with age (R_s_=0.16; *P*=0.009). In age-adjusted conditional logistic models, cases, and their matched controls were similar with respect to reproductive history, other lifestyle factors, and body measurements, except for waist circumference, where controls had somewhat smaller waists (age-adjusted *P*=0.047) (**Table 1**). The majority of patients had an invasive tumor size less than 21 mm (pT1). About half of the cases and matched controls were axillary lymph node negative and about 80% of the patients had ER^+^ tumors. Cases had somewhat more often poorly differentiated tumors (histological grade III), compared with controls, but there were no significant differences between cases and matched controls in any of the tumor characteristics.

**Table 1:** Patient- and tumor characteristics in cases and controls.

	All (n = 265)	Missing	Cases (n = 95)	Controls (n = 170)
	Median (IQR) or n (%)	(n)	Median (IQR) or n (%)	Median (IQR) or n (%)
Age at inclusion (years)	58.7 (47.8–64.2)	0	57.8 (46.3–65.3)	59.8 (48.2–64.1)
Weight (kg)	69.0 (62.0–78.8)	0	70.0 (63.0–79.8)	68.2 (61.7–78.1)
Height (m)	1.66 (1.60–1.70)	0	1.67 (1.61–1.70)	1.66 (1.60–1.70)
BMI (kg/m^2^)	25.0 (22.9–28.1)	0	25.3 (23.0–29.0)	24.9 (22.8–27.5)
Waist circumference (cm)	86 (79–95)	1	88 (81–98)	84 (77–93)
Hip circumference (cm)	102 (97–109)	1	104 (98–110)	102 (96–108)
Waist to hip ratio	0.83 (0.78–0.89)	1	0.85 (0.79–0.90)	0.82 (0.78–0.88)
Total breast volume (ml)*	1,000 (700–1,550)	34	1,100 (800–1,600)	1,000 (650–1,500)
Age at menarche (years)	13 (12–14)	1	13 (12–14)	13 (12–14)
Number of full-term pregnancies	2 (1–3)	0	2 (1–3)	2 (1–3)
Age at first full term pregnancy (years)	24 (22–28)	38	24 (21–27)	25 (22–28)
Ever oral contraceptive use	187 (70.6%)	0	67 (70.5%)	120 (70.6%)
Ever treatment for menopausal symptoms	114 (43.0%)	1	38 (40.0%)	76 (45.0%)
Current smoker	56 (21.1%)	0	24 (25.3%)	32 (18.8%)
Alcohol abstainer	27 (10.2%)	0	11 (11.6%)	16 (9.4%)
Coffee consumption ≥ 2 cups/day	215 (81.1%)	0	74 (77.9%)	141 (82.9%)
Tumor characteristics, **	n (%)		n (%)	n (%)
Invasive tumor size (pT)		0		
1 ≤ 20 mm	162 (61.1%)		55 (57.9%)	107 (62.9%)
2 21–50 mm	94 (35.5%)		35 (36.8%)	59 (34.7%)
3 ≥ 51 mm	9 (3.4%)		5 (5.3%)	4 (2.4%)
4 Skin or muscular involvment	0		0	0
Axillary nodal involvment		1		
None	140 (53.0%)		48 (51.1%)	92 (54.1%)
1–3	86 (32.6%)		30 (31.9%)	56 (32.9%)
≥ 4	38 (14.3%)		16 (17.0%)	22 (12.9%)
Histological grade		1		
I	59 (22.3%)		21 (22.1%)	38 (22.5%)
II	135 (51.1%)		40 (42.1%)	95 (56.2%)
III	70 (26.5%)		34 (35.8%)	36 (21.3%)
Homone receptor status		0		
ER^+^	216 (81.5%)		76 (80.0%)	140 (82.4%)
ER^−^	49 (18.5%)		19 (20.0%)	30 (17.6%)
PR^+^	163 (61.7%)		55 (57.9%)	108 (63.5%)
PR^−^	102 (38.5%)		40 (42.1%)	62 (36.5%)
ER^+^PR^+^	159 (60.0%)		52 (54.7%)	107 (62.9%)
ER^+^PR^−^	57 (21.5%)		24 (25.3%)	33 (19.4%)
ER^−^PR^−^	45 (17.0%)		16 (16.8%)	29 (17.1%)
ER^−^PR^+^	4 (1.5%)		3 (3.2%)	1 (0.6%)
**Hormone levels, median (IQR)**		0		
IGF-I (ng/ml)	132 (106–165)		135 (110–175)	130 (103–160)
IGFBP-3 (ng/ml)	4,290 (3,648–4,893)		4,427 (3,846–4,954)	4,261 (3,615–4,867)
IGFBP-7 (ng/ml)	35.1 (29.5–39.7)		34.7 (30.0–40.8)	35.4 (29.1–39.5)
IGF-I/IGFBP-3 molar ratio	0.111 (0.093–0.136)		0.118 (0.092–0.138)	0.110 (0.093–0.136)

*In patients without previous breast surgeries.

**In case of bilateral tumors (eight patients) the tumor characteristics for the side of the most aggressive tumor are reported.

### Preoperative Hormone Levels in Relation to Clinicopathological Characteristics

Circulating baseline IGF-I, IGFBP-3, IGFBP-7 levels or IGF-I/IGFBP-3 molar ratio were assessed in relation to selected clinicopathological characteristics among all patients and analyzed with generalized linear means models. The preoperative age-adjusted mean IGF-I level was slightly higher in patients with a history of menopausal hormone therapy (age-adjusted *P*=0.047), but was not significantly associated with overall body constitution or lifestyle factors such as smoking, alcohol or coffee consumption. However, age-adjusted preoperative IGFBP-3 levels were positively associated with body constitution. Patients with a BMI ≥25 kg/m^2^, waist circumference ≥88 cm or waist-to-hip ratio >85 had higher age-adjusted mean IGFBP-3 levels compared with leaner patients (all *P_s_*<0.032). In line with this, the age-adjusted mean IGF-I/IGFBP-3 molar ratios were significantly negatively associated with BMI, waist circumference, waist-to-hip ratio, as well as a total breast volume (all *P_s_*<0.0003). IGFBP-7 levels were not associated with any patient characteristics, **Table 2**.

**Table 2:** Age-adjusted geometric means with 95% Wald confidence intervals (CIs) of IGF-I, IGFBP-3, IGFBP-7, and IGF-I/IGBP-3 molar ratios in relation to patient and tumor characteristics.

	IGF-I		IGFBP-3		IGFBP-7		IGF-I/IGFBP-3	
	(ng/ml)	*P*-value	(ng/ml)	*P*-value	(ng/ml)	*P*-value	Molar ratio	*P*-value
**All patients**	**131 (126–136)**		**4,212 (4,113–4,312)**		**35.1 (34.1–36.2)**		**0.112 (0.109–0.116)**	
BMI < 25 kg/m^2^	135 (128–141)	0.11	4,101 (3,965–4,240)	**0.028**	35.3 (33.9–36.8)	>0.3	0.119 (0.114–0.123)	**0.0002**
BMI ≥ 25 kg/m^2^	127 (121–134)		4,326 (4,183–4,474)		34.9 (33.4–36.4)		0.106 (0.102–0.111)	
Waist circumference < 88 cm	135 (129–141)	0.083	4,115 (3,985–4,249)	**0.032**	34.7 (33.4–36.1)	>0.3	0.118 (0.114–0.123)	**0.0001**
Waist circumference ≥ 88 cm	126 (120–133)		4,343 (4,188–4,504)		35.4 (33.8–37.1)		0.105 (0.101–0.110)	
Waist to hip ratio ≤ 0.85	133 (127–139)	>0.3	4,082 (3,958–4,211)	**0.002**	35.2 (33.8–36.6)	>0.3	0.117 (0.113–0.122)	**0.0002**
Waist to hip ratio > 0.85	129 (122–135)		4,401 (4,244–4,565)		34.8 (33.3–36.5)		0.105 (0.101–0.110)	
Total breast volume < 850 ml*	138 (129–146)	0.066	4,057 (3,890–4,231)	0.083	35.1 (33.2–37.1)	>0.3	0.122 (0.116–0.129)	**0.0003**
Total breast volume ≥ 850 ml*	128 (123–134)		4,249 (4,121–4,381)		35.0 (33.6–36.5)		0.109 (0.105–0.113)	
Ever OC use, no	129 (120–137)	>0.3	4,193 (4,002–4,393)	>0.3	35.1 (33.1–37.2)	>0.3	0.111 (0.109–0.117)	>0.3
Ever OC use, yes	132 (127–138)		4,219 (4,098–4,343)		35.1 (33.9–36.4)		0.113 (0.109–0.117)	
Ever MHT, no	127 (121–133)	**0.047**	4,163 (4,030–4,301)	0.30	35.9 (34.5–37.4)	0.13	0.110 (0.106–0.114)	0.14
Ever MHT, yes	137 (129–144)		4,277 (4,119–4,442)		34.2 (32.6–35.8)		0.115 (0.110–0.121)	
Current smoker, no	131 (126–136)	>0.3	4,247 (4,135–4,361)	0.18	34.9 (33.8–36.1)	>0.3	0.111 (0.108–0.115)	0.23
Current smoker, yes	131 (122–141)		4,082 (3,877–4,298)		35.7 (33.5–38.1)		0.116 (0.109–0.124)	
Alcohol abstainer, no	132 (127–137)	>0.3	4,233 (4,128–4,340)	0.21	35.0 (33.9–36.1)	>0.3	0.112 (0.109–0.122)	>0.3
Alcohol abstainer, yes	125 (112–139)		4,027 (3,738–4,339)		36.4 (33.2–40.0)		0.112 (0.102–0.122)	
Coffee 0–1 cups/day	132 (122–142)	>0.3	4,273 (4,139–4,620)	0.14	34.5 (32.2–37.0)	>0.3	0.109 (0.102–0.116)	0.28
Coffee ≥ 2 cups/day	131 (126–136)		4,174 (4,066–4,286)		35.3 (34.1–36.4)		0.113 (0.110–0.117)	
Tumor characteristics, **								
Invasive tumor size (pT)								
≤ 20 mm (1)	135 (129–141)	**0.040**	4,197 (4,071–4,327)	>0.3	35.3 (34.0–36.6)	>0.3	0.116 (0.112–0.120)	**0.006**
≥ 21mm (≥ 2)	125 (119–132)		4,233 (4,075–4,398)		34.8 (33.2–36.6)		0.107 (0.102–0.112)	
Axillary nodal involvment								
None	130 (125–137)	>0.3	4,287 (4,149–4,429)	0.30	35.0 (33.6–36.4)	>0.3	0.110 (0.106–0.114)	0.28
1–3	131 (123–139)		4,115 (3,947–4,290)		34.8 (33.0–36.7)		0.115 (0.109–0.121)	
≥ 4	133 (122–146)		4,165 (3,911–4,435)		35.9 (33.2–38.8)		0.116 (0.107–0.125)	
Histological grade								
I	132 (123–142)	>0.3	4,112 (3,909–4,324)	>0.3	34.2 (32.1–36.5)	>0.3	0.116 (0.109–0.124)	>0.3
II	130 (124–136)		4,240 (4,101–4,384)		34.8 (33.4–36.3)		0.111 (0.106–0.115)	
III	132 (123–141)		4,244 (4,051–4,446)		36.4 (34.3–38.5)		0.112 (0.106–0.119)	
Homone receptor status								
ER^+^	132 (127–137)	0.22	4,243 (4,133–4,356)	0.19	35.0 (33.8–36.1)	>0.3	0.113 (0.109–0.116)	>0.3
ER^−^	125 (116–136)		4,073 (3,854–4,306)		35.8 (33.4–38.3)		0.111 (0.104–0.119)	
PR^+^	132 (126–137)	>0.3	4,158 (4,034–4,286)	0.18	34.8 (33.5–36.1)	>0.3	0.114 (0.110–0.119)	0.14
PR^−^	130 (123–137)		4,297 (4,136–4,465)		35.7 (34.0–37.4)		0.109 (0.104–0.114)	

*In patients without previous breast surgeries.

**In case of bilateral tumors (eight patients) the tumor characteristics for the side of the most aggressive tumor are reported.

Bold values indicate significant P-values < 0.05.

Patients with larger invasive tumors (≥21 mm) had lower age-adjusted IGF-I levels and IGF-I/IGFBP-3 molar ratio compared with those with tumors ≤20 mm (age-adjusted *P*=0.040 and *P*=0.006, respectively). No other associations between tumor characteristics and IGF-I, IGFBP-3, IGFBP-7 levels, or IGF-I/IGFBP-3 molar ratios were found, **Table 2**.

### Risk of Breast Cancer Recurrence in Relation to Preoperative IGF-I, IGFBP-3, IGFBP-7, or IGF-I/IGFBP-3 Levels

**Figure 2:**
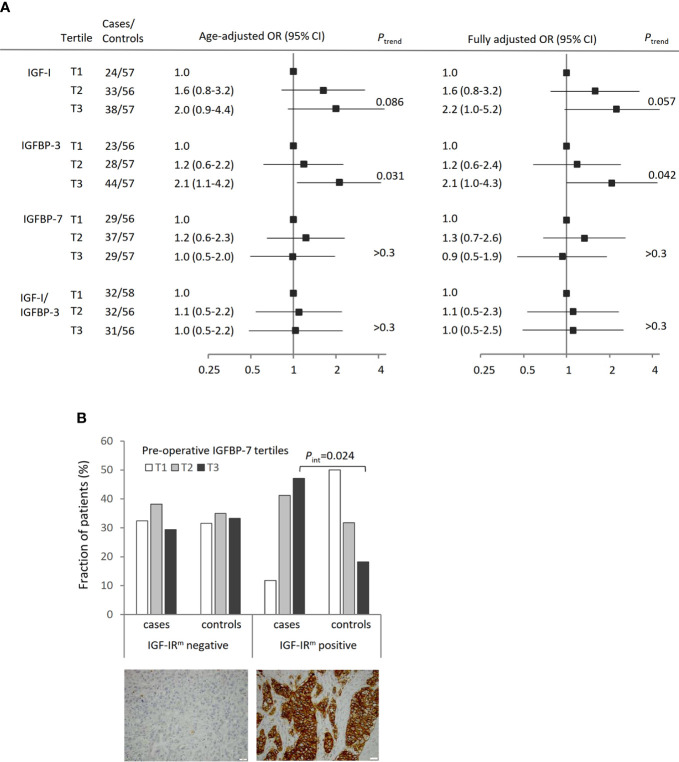
Preoperative systemic hormone levels in relation to breast cancer recurrence. **(A)** Conditional odds ratios (OR) with 95% confidence interval (CI) of breast cancer recurrence according to tertiles of IGF-I, IGFBP-3, IGFBP-7 levels and IGF-I/IGFBP-3 molar ratio, adjusted for age. The fully adjusted model is adjusted for age, BMI ≥25 kg/m^2^, waist circumference ≥88 cm, preoperative smoking, and treatments. One control had a missing waist measurement and is excluded from the fully adjusted model but included in the total number for IGF-I (T2), IGFBP-3 (T1), IGFBP-7 (T3) and IGF-I/IGFBP-3 molar ratio (T3). The lowest tertile was used as reference. **(B)** Distribution of cases with breast cancer recurrence (n=85) or recurrence-free controls (n=139) in relation to IGFBP-7 tertiles and IGF-IR membrane (IGF-IR^m^) negative or positive primary tumors. There was a significant interaction between IGFBP-7 T3 levels and IGF-IR^m^ positivity on recurrence-risk (*P*_interaction_=0.024).

The risk of breast cancer recurrence was evaluated in relation to preoperative low (T1), moderate (T2), or high (T3) tertile IGF-I, IGFBP-3, IGFBP-7 levels, or IGF-I/IGFBP-3 molar ratios between cases and matched controls (**Figure 2A**). The preoperative IGF-I and IGFBP-3 levels were prognostic indicators of breast cancer recurrence. High age-adjusted IGF-I (T3: OR 2.00, 95% CI 0.92–4.37; *P*_trend_=0.086) as well as high age-adjusted IGFBP-3 levels (T3: OR 2.10, 95% CI 1.06–4.15; *P*_trend_=0.031) were associated with higher odds of breast cancer recurrence compared with low IGF-I or IGFBP-3 levels. No independent associations between IGFBP-7 or IGF-I/IGFBP-3 tertiles and breast cancer recurrence were observed (*P*_trends_>0.3).

With further adjustments for BMI (≥25 kg/m^2^), waist circumference (≥88 cm), current smoking at the preoperative visit, and ever tamoxifen-, aromatase inhibitor-, radiation-, or chemotherapy treatment prior to recurrence in the case and by the corresponding follow-up time in the matched control, the *P*_trend_-values were similar with increasing recurrence-risk by increasing preoperative levels of IGF-I (*P*_trend_=0.057) or IGFBP-3 (*P*_trend_=0.042), while there was no association for IGFBP-7 or the IGF-I/IGFBP-3 molar ratio.

Given the reported biological significance of interactions between IGF-I, IGFBP-7, and IGF-IR, correlations between preoperative circulating IGF-I, IGFBP-3, and IGFBP-7 levels, and tumor IGF-IR membrane (IGF-IR^m^) expression were assessed. Among the included patients with available TMA tissue for both the case (n=85) and at least one matched control (n=139), 17 cases and 22 controls were IGF-IR^m^ positive, and 68 cases and 117 controls were IGF-IR^m^ negative (**Figure 2B**).

No prognostic associations between preoperative IGF-I or IGFBP-3 levels and IGF-IR^m^ expression were found. However, the IGFBP-7 levels among patients with IGF-IR^m^ positive tumors were inversely distributed among cases with breast cancer recurrence and recurrence-free controls (**Figure 2B**). Among patients with positive IGF-IR^m^ expression, increasing IGFBP-7 levels were associated with poorer prognosis, with an age-adjusted OR of 3.47 (95% CI 0.44–27.47) for IGFBP-7 T2 while for IGFBP-7 T3 the age-adjusted OR was 10.20 (95% CI 1.36–76.57). The interaction between IGFBP-7 T3 levels and IGF-IR^m^ positivity on recurrence-risk was significant (*P*_interaction_=0.024).

### Postoperative Changes in Plasma Analyte Levels in Relation to Clinicopathological Characteristics and Type of Adjuvant Treatment

**Figure 3:**
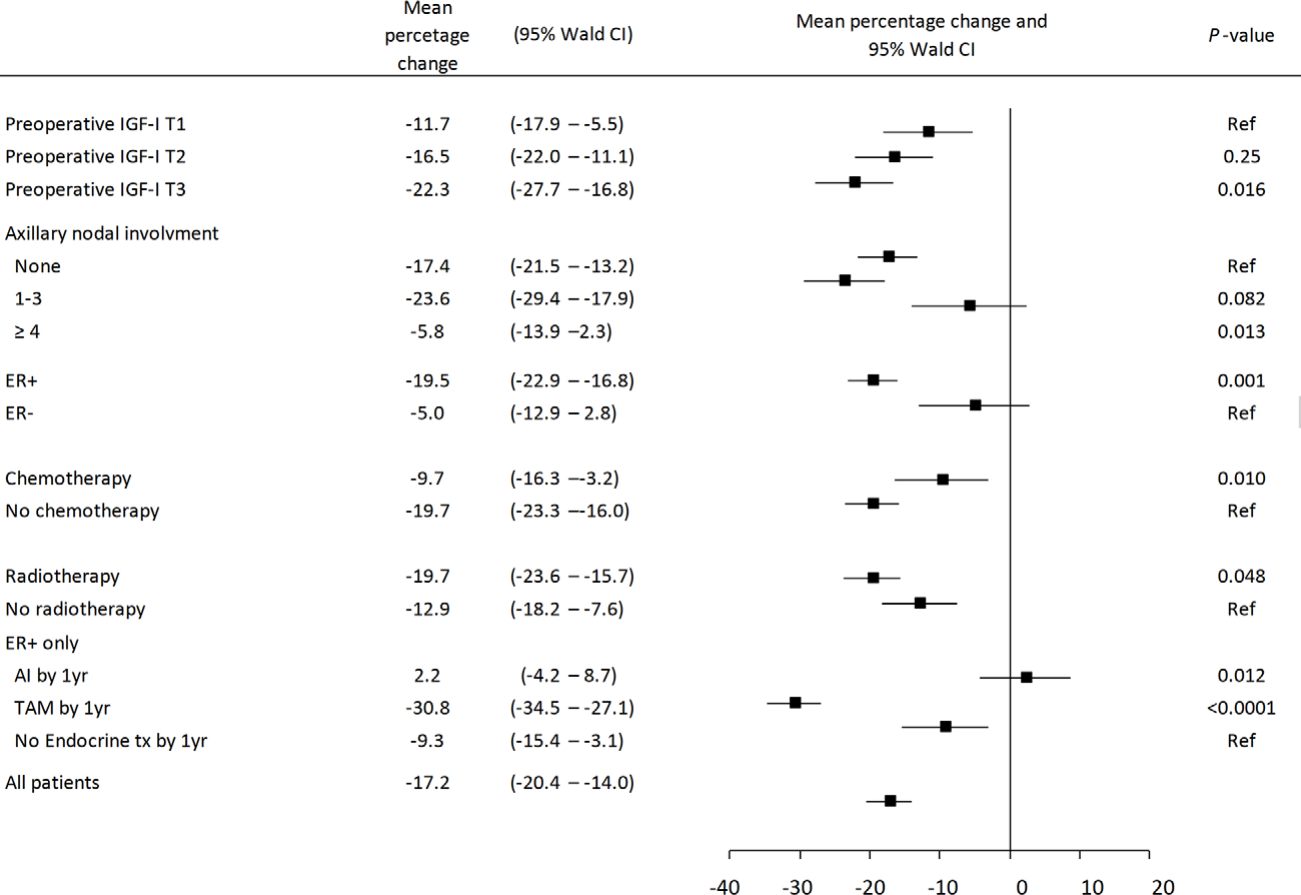
Mean percentage change in IGF-I levels with 95% Wald CIs between the preoperative and 1-year postoperative samples, adjusted for age. No significant differences were seen in the percentage change depending on invasive tumor size, histological grade or PR status. Chemotherapy was no longer independently significantly associated with percentage change in IGF-I levels in a multivariable model including all factors.

Postoperative changes in IGF-I, IGFBP-3, IGFBP-7, or IGF-I/IGFBP-3 levels were assessed in the 208 patients with both pre- and postoperative analyte levels. Overall, the age-adjusted IGF-I levels were reduced by 17.2% (95% Wald CI −20.4 to −14.0%) 1-year post surgery compared with the preoperative levels (**Figure 3**). The reduction in IGF-I levels was more pronounced the higher the preoperative IGF-I tertile (*P*_trend_=0.017) in patients with ER^+^ tumors and in patients with 1–3 positive axillary lymph nodes, but not in patients with ≥4 positive lymph nodes. Invasive tumor size, histological grade, and PR status were not associated with changes in IGF-I levels. Patients were not matched on therapy. For controls, only the adjuvant treatments that were used prior to the time of recurrence in the matched case were considered and all treatments after that time-point were censored. For the 208 patients, 24.7% of the cases had undergone chemotherapy *versus* 18.8% of the controls (*P*=0.011), and 60.0% of the cases had undergone radiotherapy *versus* 66.2% of the controls (*P*>0.3). For the 175 patients with ER^+^ tumors, 40% of the cases had used tamoxifen *versus* 56.4% of the controls by the 1-year visit (*P*=0.023). AIs had been used by 22.7% of the cases *versus* 14.3% of the controls (*P*=0.12) by the 1-year visit.

With respect to treatments, IGF-I levels were more reduced in patients who were chemonaïve or had been treated with radiotherapy compared with other patients. In patients with ER^+^ tumors, the type of endocrine therapy impacted the magnitude of the change and a greater reduction was seen in tamoxifen-treated patients compared with patients with no endocrine therapy (*P*<0.0001), while AI-treated patients had a non-significant increase in their IGF-I levels by the 1-year visit. In a multivariable model, chemotherapy was no longer statistically significantly associated with changes in IGF-I levels.

Both IGFBP-3 and IGFBP-7 levels were relatively stable between the preoperative and 1-year postoperative samples, regardless of tumor characteristics and treatments. IGFBP-3 levels in AI-treated patients were slightly higher at the 1-year postoperative visit compared with preoperative levels (*P*=0.048) while IGFBP-7 levels in patients with invasive tumor sizes ≥21 mm were slightly reduced at the 1-year postoperative visit compared with preoperative levels (*P*=0.024). No other statistically significant changes were observed.

### Multivariable Models of Preoperative Analyte Levels and Postoperative Changes in Relation to Recurrence

The prognostic role of postoperative changes in IGF-I, IGFBP-3, IGFBP-7, or IGF-I/IGFBP-3 levels in relation to breast cancer recurrence was assessed. The analyte levels were considered stable if they were within 10% of the preoperative value, otherwise they were considered increased or reduced.

The postoperative changes in the plasma IGF-I levels were associated with an increased recurrence-risk in the model adjusted for age- and preoperative IGF-I tertiles, but not in the fully adjusted model where BMI, waist circumference, preoperative current smoking, and adjuvant treatments were taken into account (**Table 3**). By the time of recurrence in the case, tamoxifen had been used by 56.5% of the cases *versus* 72.6% of the matched controls (*P*=0.030) and AIs had been used by 38.7% of the cases *versus* 40.7% of the matched controls (*P*>0.3). Postoperative changes in other analyte levels were not predictive of breast cancer recurrence when adjusting for age, BMI, waist circumference, preoperative current smoking, tamoxifen-, AI-, radiation-, and chemotherapy treatment.

**Table 3:** Multivariable conditional logistic regression models of the impact of percentage change in IGF-I levels between the preoperative and 1-year visits on recurrence in 208 patients with measurements from both visits.

	Cases/controls		Model 1		Model 2	
			OR (95% CI)	*P-*value	OR (95% CI)	*P-*value
**IGF-I reduction (>10%)**	40/93		1.00		1.00	
**IGF-I stable (max ±10% change)**	22/28		1.81 (0.88–3.73)	0.11	1.61 (0.67–3.88)	0.29
**IGF-I increase (>10%)**	13/12		2.67 (1.09–6.58)	0.032	1.98 (0.73–5.57)	0.18
		*P*_trend_		0.015		0.15

Model 1: adjusted for preoperative levels and age.

Model 2: adjusted for preoperative levels, age, BMI ≥ 25 kg/m^2^, waist circumference ≥ 88 cm, current smoking at preoperative visit, chemotherapy, radiotherapy, tamoxifen, aromatase inhibitor by time of recurrence in the matched case.

## Discussion

There is prior evidence that systemic IGF-I and IGFBP-3 are associated with risk of breast cancer (4, 11), but IGFBP-7 has not been studied in this context. Furthermore, associations between pre- and postoperative levels of circulating IGF-I, IGFBP-3, and IGFBP-7 and breast cancer recurrence have not yet been studied. The present study demonstrates that preoperative levels of IGF-I or IGFBP-3 (tertiles) are positively associated with risk of breast cancer recurrence, which is in contrast to a recent study of Danish postmenopausal patients (28). Meanwhile, postoperative changes in IGF-I, IGFBP-3, IGFBP-7, or IGF-I/IGFBP-3 molar ratios were not prognostic indicators of breast cancer recurrence once treatment was taken into account. Tamoxifen-treatment of patients with ER^+^ tumors substantially reduced systemic IGF-I levels among both cases and controls, confirming previous reports (29). Only AI-treatment was associated with relatively stable IGF-I levels and others have reported no statistically changes in either IGF-I concentrations or the IGF-I/IGFBP-3 molar ratio over the first 6 months (30), although on small study of two groups of 15 patients with advanced breast cancer who received either 0.5 or 2.5 mg letrozole reported a short-term increase in IGF-I but not IGFBP-3 levels during the first 3 months of treatment that was independent of dose (31). Finally, patients who had received radiotherapy had a significantly larger reduction in IGF-I levels compared to the rest of the patients independent of other treatments, and this has to our knowledge not been previously reported.

In the present Swedish cohort, increasing pre-operative circulating levels of IGF-I were associated with higher odds of breast cancer recurrence. A recent Chinese study, found no association between pre-operative levels of IGF-I, IGFBP-3, or IGF-I/IGFBP-3 ratios and breast cancer recurrence (32). The inter-individual variation in systemic IGF-levels is influenced by age, menstrual cycle phase, genetic factors, lifestyle (33–35), and body size (36, 37). Most patients in the present study were postmenopausal and therefore no adjustment for cycle phase was carried out in the fully adjusted model, instead we adjusted for age. Further it was hard to determine exact age at menopause since younger patients with progestin-containing intra uterine devices or hysterectomies without oophorectomies reported that they did not have menstrual periods. In contrast, older postmenopausal patients on MHT reported their last hormone induced bleeding as their last period. While higher IGF-I levels were only weakly associated with smaller body sizes, higher preoperative IGF-I/IGFBP-3 molar ratios were strongly associated with smaller body sizes including breast volume, which to our knowledge has not been reported before. A recent study based on the BCBlood cohort showed that a breast volume ≥850 ml was the strongest prognostic anthropometric factor in terms of breast cancer-free interval, both in all patients and in various treatment groups. Meanwhile, BMI and waist circumference were the strongest prognostic anthropometric factors for overall survival (38). Given the inverse association between the body size and the IGF-I/IGFBP3 molar ratio, it is unlikely that the association between breast volume and recurrence-risk is mediated *via* increases in IGF-I or IGFBP-3.

In the present study we found that the majority of patients had reduced IGF-I levels by the 1-year visit. The magnitude of reduction in IGF-I levels was mainly dependent on the preoperative IGF-I tertile, ER status, radiotherapy, and tamoxifen treatment, but not with AI-treatment or changes in body size. Mason et al. have reported that the relationship between weight change and IGF-I or the IGF-I/IGFBP3 molar ratio is complex and differs depending on type of weight loss, (diet or diet+exercise) (39). Postoperative weight change of >5% during the first postoperative year did influence the recurrence-risk in this cohort (40). However, changes in IGF-I levels were not associated with overweight at the preoperative visit or >5% change in body weight between the visits (data not shown). Further, postoperative changes in IGF-I levels were not independently associated with recurrence-risk.

The reported pleiotropic roles of IGFBP-7 suggest a potential switch in biological properties with tumor progression, similar to that of TGF-β. Low tumor IGFBP-7 levels have been associated with poor prognosis in gastric cancer (41) and breast cancer (23). In contrast, positive IGFBP-7 expression in adenocarcinoma was associated with significantly reduced median and 5-year survival (42). Circulating IGFBP-7 levels overall were not an independent predictor of breast cancer recurrence in the present study cohort. However, among patients with IGF-IR^m^ positive primary tumors, increasing preoperative IGFBP-7 levels were associated with higher odds of breast cancer recurrence, while no associations between IGFBP-7 levels and breast cancer recurrence were observed among patients with IGF-IR^m^ negative primary tumors. The interaction between circulating IGFBP-7 and activated tumor-specific IGFIR/InsR, analyzed in a previous study (27), was of lower magnitude and did not reach statistical significance (data not shown). However, the number of patients in some subgroups was small. These results therefore need to be interpreted with caution and validated in an independent study. Modulation of IGF signaling by IGFBPs is complex. IGFBPs simultaneously increase IGF-I half-life while reducing availability for receptor binding, which means more IGF-I present but presumably with reduced activity; and the final effect is further modulated by presence or absence of the IGFBP proteases, which release the free IGFs from the IGFBP complex (1). This study focused on two of the IGFBPs, i.e., IGFBP-3 and IGFBP-7, since IGFBP3 is crucial for estimation of the biologically active fraction of IGF-I. In addition we choose to study IGFBP-7, which is less well evaluated in the breast cancer setting. Other BPs such as IGFBP-1, IGFBP-2, and IGFBP-5 also play a role in breast cancer (18), but were outside the scope of this study. The role of IGFBP-7 in modulating IGF-IR activity in breast cancer merits further study.

This nested case control study was done within the BCBlood cohort, a population-based well-annotated cohort with excellent follow-up (43). Matching was done by sampling prevalent cases with recurrence that were matched to one or two recurrence-free controls. On the one hand, this method may potentially result in too healthy controls. On the other hand, selected controls are more likely to resemble cases if matched on tumor characteristics and may therefore have more aggressive cancers than those in unselected controls from the same cohort. We matched on age and tumor characteristics to enable study of the independent effects of the IGF analytes and to reduce confounding. Although cases and controls were matched on age, we allowed for up to five years difference between the case and each matched control. Since all analytes were strongly correlated with age, age was retained as a covariate in all models and age-adjusted numbers are presented in order to minimize residual confounding. Gene expression profiling, such as MammaPrint or OncotypeDx, is not yet used in clinical routine in Sweden and molecular subtypes were thus not available, why we were unable to assess whether any associations found were independent of molecular subtypes. HER2 was not routinely analyzed prior to Nov 2005, thus missing for large number of patients and not included in the present study. Cases and controls were not matched on treatment, why we chose to adjust for treatment in the analyses of postoperative changes. Another matching method would have been to use incidence density sampling where a patient could first be a control and then a case and thus be counted twice (44). Since discrepancies between studies in the IGF-field may in part be attributed to use of different test methods, the analytic assays selected for this study have been well validated (45) and all samples were run in duplicate. The antibody used for IGF-IR was also carefully selected (27) in order to minimize non-specific binding.

In conclusion, this nested case-control study provides evidence that preoperative, but not postoperative IGF-I and IGFBP-3 levels provide independent prognostic information and influence breast cancer recurrence, justifying confirmatory studies with larger numbers of subjects. The role of systemic IGFBP-7 and its interplay with the IGF-IR in breast cancer merit further study. Further understanding of the prognostic and treatment predictive role of the IGF system in breast cancer progression and clinical outcome is needed in order to determine its therapeutic potential.

## Data Availability Statement

The datasets generated for this study will not be made publicly available due to privacy laws. Questions regarding data should be directed to helena.jernstrom@med.lu.se

## Ethics Statement

The study was reviewed and approved by Lund university ethics committee. The patients/participants provided their written informed consent to participate in this study.

## Author Contributions

AR and HJ: study design and manuscript preparation. HJ, CI, and SBj: study supervision, AR, SBj, KJ, MP, and HJ: data collection and analyses of markers. AR and HJ: statistical analysis, data analysis, and interpretation. KJ, SB, CI, and MP: contributed to manuscript review and critical revision for important intellectual content, and read and approved the final draft for submission. All authors contributed to the article and approved the submitted version.

## Funding

This study was supported by grants from the Swedish Cancer Society, the Faculty of Medicine at Lund University, the South Swedish Health Care Region (Region Skåne ALF), the Skåne University Hospital Fund, the Swedish Breast Cancer Group (BRO), the Mrs. Berta Kamprad Foundation, the Gunnar Nilsson Foundation, King Gustaf V’s Jubilee Foundation, The Percy Falk foundation, and the Royal Physiographic Society in Lund.

## Conflict of Interest

The authors declare that the research was conducted in the absence of any commercial or financial relationships that could be construed as a potential conflict of interest.

## Abbreviations

AI, aromatase inhibitor; BMI, body mass index; CI, confidence interval; ELISA, enzyme-linked immunosorbent assay; EMT, epithelial-to-mesenchymal; ER, estrogen receptor; IGF-I, insulin-like growth factor (IGF)-I; IGFIR, insulin-like growth factor I receptor; IGF-IR^m^, membranous insulin-like growth factor I receptor; IGFBP-3, insulin-like growth factor-binding protein 3; IGFBP-7, insulin-like growth factor-binding protein 7; IGFBPrp1, insulin-like growth factor-binding protein-related protein 1; MHT, menopausal hormone therapy; OR, odds ratio; PR, progesterone receptor; REF, reference; RXR-α, nuclear retinoid X receptor α; T, tertile; TMA, tissue microarrays.
